# Levels and predictors of postpartum depression and anxiety during the first year of the COVID-19 pandemic in a confined cross-border city

**DOI:** 10.1007/s12144-023-04719-6

**Published:** 2023-05-10

**Authors:** Alicia Remartínez-Hamed, María Ángeles Pérez-Morente, María Adelaida Álvarez-Serrano, Encarnación Martínez-García, Alberto González-García, Inmaculada García-García, Adelina Martín-Salvador

**Affiliations:** 1 Department of Nursing, Faculty of Health Sciences, University of Granada, 18016 Granada, Spain; 2 Department of Nursing, Faculty of Health Sciences, University of Jaén, 23071 Jaén, Spain; 3 Department of Nursing, Faculty of Health Sciences, University of Granada, St. Cortadura del Valle S/N, 51001 Ceuta, Spain; 4 Guadix High Resolution Hospital, 18500 Granada, Spain

**Keywords:** Postpartum, Depression, Anxiety, Mood disorders

## Abstract

Pregnancy and/or the puerperium involve social, physiological and psychological changes that make women more vulnerable to mental disorders such as anxiety and depression, even more so if these develop in stressful contexts such as the pandemic. The aim of this study is to identify factors associated with the risk of postpartum anxiety and depression during the COVID-19 pandemic. A cross-sectional study was conducted among postpartum women (*n* = 69) who gave birth between March 2020 and March 2021 in Melilla, a Spanish cross-border city with Morocco whose borders were closed, making it a confined city. The scales used were the State-Trait Anxiety Inventory and the Edinburgh Postnatal Depression Scale. Results reported an elevated risk of depression (85.5%) and anxiety (63.8%), with severe anxiety reaching 40.6% of cases. Predictors of postpartum depression comprised a personal history of mood disorders (*β* = 8.421; CI95% = 4.863/11.978) and having been diagnosed with COVID-19 during pregnancy or postpartum (*β* = 4.488; CI95% = 1.331/7.646). As regards anxiety, it is predicted on the basis of mood antecedents (*β* = 14.175; CI95% = 7.870/20.479), the fact of having been diagnosed with COVID-19 during pregnancy or postpartum (*β* = 8.781; CI95% = 2.970/14.592) and the fact of being a multipara (*β* = 5.513; CI95% = 0.706/10.321). In conclusion, special attention should be paid to women with a history of mood disorders and a positive diagnosis of COVID-19 during pregnancy or postpartum, even more so in the case of multiparous women, because of its impact on mental health during the postpartum period.

**Supplementary Information:**

The online version contains supplementary material available at 10.1007/s12144-023-04719-6.

## Introduction

Pregnancy and postpartum periods are characterised for being periods in which women experience many changes at social, physiological, and psychological levels (Lutkiewicz et al., 2020), which may lead to the appearance or relapse of mental disorders such as depression and anxiety (Biaggi et al., 2016). These two disorders affect both the mother’s and the new-born’s health and even the relationship between them (Mohammad-Redzuan et al., 2020; Biaggi et al., 2016; Branquinho et al., 2020).

Pursuant to the American Psychiatric Association (APA, 2014) postpartum depression (PPD) is the most frequent mental disorder during the puerperium. It is defined as a major depressive episode which may start during pregnancy or during the first four weeks following the postpartum period and which may persist until a year after delivery. It is characterised by sadness, anhedonia, fatigue, low self-esteem, sense of guilt, sleep disorders, decreased appetite, worries about the baby’s health, and suicidal ideation (Missler et al., 2020). On the other hand, postpartum anxiety is characterised by excessive and persistent distress related to disquiet, irritability, nervousness, fear, and sleep disorders. It is connected to PPD, and may lead to a lower possibility of breastfeeding, an increase in the risk of child abuse, new-born’s cognitive and social impairment, and a higher probability of anxiety in the baby (Mohammad-Redzuan et al., 2020).

PPD is present in 10-15% of women, particularly in less developed countries. On the other hand, postpartum anxiety affects between 8% and 13% (Molgora & Accordini, 2020) and the comorbidity of both is approximately 75% (Mohammad-Redzuan et al., 2020).

Although the exact causes remain unknown, multiple factors could influence the onset of PPD and anxiety. On the one hand, biological factors such as hormonal changes after childbirth (Molgora & Accordini, 2020). On the other hand, social and cultural factors, such as a history of child abuse (Biaggi et al., 2016), lack of social/family support during pregnancy and/or postpartum (Yan et al., 2020), poor relationship (Biaggi et al., 2016), not being married (Stepowicz et al., 2020), belonging to a low socioeconomic level (Missler et al., 2020), or being a smoker and using drugs (Spinola et al., 2020). There is also evidence of the influence of obstetric factors, for example, being multiparous (Stojanov et al., 2020), having a negative behaviour towards the pregnancy, or the type of delivery, so that there is a relationship between caesarean delivery and higher levels of PPD and anxiety (Mohammad-Redzuan et al. al., 2020). Finally, psychological factors have also been highlighted, for example, having low self-esteem and being emotionally unstable (Biaggi et al., 2016), a family or personal history of bipolar disorder, anxiety, or depression (Missler et al., 2020; Spinola et al., 2020), and serious illness, bereavement, miscarriage, and previous high-risk pregnancies (Yan et al., 2020). Having these disorders during pregnancy carries obstetric and perinatal health risks for the mother and foetus, such as an increased likelihood of pre-eclampsia, hypertension, and gestational diabetes (Stojanov et al., 2020). It is affirmed that such disorders are related to low scores on the Apgar test, prematurity, congenital disorders, low birthweight, reduction of head circumference, higher probability of suffering from depression, anxiety, attention deficit disorder, and hyperactivity in the future. PPD is also linked to disorders in the cognitive, physical, and linguistic development of children during the first year of life (Do et al., 2018; Lebel et al., 2020).

The pandemic has generated in the population an increase in the levels of fear, depression, anxiety, and stress (Stojanov et al., 2020). The prevalence of symptoms of depression and anxiety during the pandemic in the Spanish population was 18.7% and 21.6%, respectively (González-Sanguino et al., 2020). Several authors conclude that stressful contexts increase the prevalence of mental disorders in women during pregnancy and the postpartum period. In this sense, the global COVID-19 pandemic also seems to have had an impact on higher levels of anxiety and postpartum depression (Stepowicz et al., 2020; Yan et al., 2020; Lebel et al., 2020; Romero-González et al. 2020). Specifically in Spain, it has been quantified that the symptoms of anxiety and depression among pregnant women increased by 59% and 38%, respectively, during confinement (Brik et al., 2021).

Measures implemented by health services to control infection have directly affected the psychological well-being of pregnant women. These measures include spacing obstetric appointments, introducing online medical consultations or online childbirth preparation classes, and even being unaccompanied during labour. Indeed, lack of support during childbirth is one of the variables most frequently associated with the occurrence of PPD (Stojanov et al., 2020; Yan et al., 2020; Bertholdt et al. 2020; Nguyen et al., 2022).

Romero-González et al. (2020) state that fear of contagion, solitude, and stress experimented by pregnant women during the pandemic are risk factors for the development of PPD and anxiety. Likewise, during pregnancy, women are more prone to infections, which makes them a vulnerable group in the case of COVID-19. Such fact, associated with a potential transmission of the virus to the foetus, has generated a higher level of anxiety, depression, and stress in women (Mariño-Narváez et al., 2021). Additionally, García-León et al. (2022) stated that the youngest pregnant Spanish women in the first quarter of pregnancy were the most vulnerable to the effects of stress and concerns about COVID-19. However, the fear of contagion made pregnant women one of the most aware groups and predisposed to get vaccinated, even willing to pay for the vaccine if necessary (Nguyen et al., 2021).

Based on previous research on risk factors for the development of PPD and anxiety, the following hypotheses were established:

1. The risk of suffering PPD and/or anxiety differs between those who gave birth before and after the COVID-19 pandemic.
2. The risk of suffering PPD and/or anxiety differs according to sociodemographic characteristics, such as age, relationship status, religion, level of education and working status.
3. The risk of PPD and/or anxiety differs between women who have help raising their children and those who do not.
4. The risk of PPD and/or anxiety differs between those who have a history of mood disorders and those who do not.
5. The risk of PPD and/or anxiety differs between new and multiparous mothers.
6. The risk of suffering PPD and/or anxiety differs among those who have maintained close contact with or have been infected by COVID-19 and those who do not.

The objectives of this study were to identify the level of anxiety and the risk of postpartum depression during the pandemic, related factors, and predictive factors.

## Materials and Methods

### Study Design and Procedure

Cross-sectional study through an online, voluntary, anonymous, and self-administered questionnaire on women who gave birth at the Regional Hospital of the Autonomous City of Melilla (Spain) between March 2020 and March 2021, and who understood the Spanish language.

Contact with women was possible through the different health centres and lactation associations of the Autonomous City of Melilla, as well as the social media in the city, whereby means of links and QR codes, they could access the questionnaire. From the beginning, they were informed of the objectives of the study and their participation was required, as well as their informed consent. Sampling was non-probabilistic by convenience.

### Measurement Tool

To collect the data, a three-block questionnaire was developed: socio-demographic, clinical and pandemic-related characteristics, the Edinburgh Postnatal Depression Scale (EPDS), and the Spielberger State-Trait Axiety Inventory. First, socio-demographic, clinical and pandemic-related characteristics were included. Second, the Edinburgh Postnatal Depression Scale (EPDS); and third, the Spielberger State-Trait Anxiety Inventory were employed.

#### Sociodemographic, Clinical, and Pandemic-Related Characteristics

Sociodemographic variables have been collected (age, marital status, religion, education level, working status) as well as clinical variables (parity, maternal education course attendance, number of maternal education classes attended, positive COVID-19 diagnosis during pregnancy and the postpartum period, close contact with someone with COVID-19 during pregnancy and the postpartum period, personal mood antecedents). Difficulties found in the follow-up of pregnancy and the postpartum period during the pandemic (not being accompanied to medical consultations; delay in testing; delay in attention; online consultation), and the help received for the care of the new-born, have also been taken into account.

#### Postnatal Depression

The Edinburgh Postnatal Depression Scale was created by Cox, Holden, and Sagovsky in 1987, with the aim of helping primary care health professionals to detect PPD. The split-half reliability of the scale was found to be 0.88, and the standardised a-coefficient 0.87 (Cox, Holden, & Sagovsky, 1987). This questionnaire validated in Spanish consists of 10 short questions to know mothers’ mood during the last 7 days. Each question has a score between 0 and 3 using a 4-point Likert scale. Items assessed as reversed are 3, 5, 6, 7, 8, 9, and 10. Total score varies between 0 and 30, 10 being the cut-off value, which indicates risk of suffering from said disorder, 0 to 10 being deemed with no risk, and 10 to 30 being deemed risk of PPD (Krauskopf & Valenzuela, 2020).

#### Postnatal Anxiety

The State-Trait Anxiety Inventory was created by Spielberger, et al. in 1983. It is the self-administered questionnaire mostly employed by Spanish psychologists to assess anxiety. It consists of 40 items, divided into two subscales with 20 items to assess trait anxiety (STAI-R) and 20 items to assess state anxiety (STAI-E) using a 4-point Likert scale running from 4 (rarely or nothing) to 3 (almost always or often) in trait and state anxiety, respectively. Updated by Guillén-Riquelme & Buela-Casal (2011), Cronbach’s alpha reliability was 0.90 for Trait and 0.94 for State Anxiety. Reversed items are considered for STAI-R (21,26,27,30,33,36, and 39) and for STAI-E (1,2,5,8,10,11,15,16,19, and 20). For this study, a simplified version of this questionnaire was utilised, the STAI-E, which assesses the level of anxiety generated by a stressful episode, such as the postpartum period, as used by other authors (Gancedo-García et al., 2017; Navas-Arrebola et al., 2022). Total score fluctuates from 0 to 60, including no anxiety (<20 points), mild anxiety (20-25 points), moderate anxiety (26-32 points) and serious anxiety (33-60 points) (Gancedo-García et al., 2017).

### Data Analysis

Data analysis was performed using the Statistical Package for the Social Sciences (SPSS) program, version 22, (IBM, New York, NY, USA, for Windows). After data cleaning and coding, the specific analytical procedures were as follows:

1. The demographic characteristics of the participants were identified using descriptive statistics for the whole sample, calculating frequencies and percentages for qualitative variables and measures of central tendency (mean and standard deviation) for quantitative variables. To check the normality of the sample, the Kolmogorov-Smirnov and Shapiro-Wilk tests were used.
2. Bivariate analyses were conducted for depression and anxiety, the sociodemographic variables (age, level of education, marital status, and working status), other variables (number of children, maternal education, mood antecedents, and having had help during the postpartum period to take care of the new-born) and variables related to the COVID-19 pandemic (COVID-19 diagnosis and close contact with the virus) being deemed the independent variables. The Pearson Correlation Coefficient was applied to calculate the relationships of the total of depression and anxiety risk scales with the age and number of children, and the Student’s T Test was applied for the relationship of the total of scales with the remaining dichotomous variables utilised in the study. Furthermore, the chi-squared test was conducted to associate the classified scores of the depression and anxiety risk scales with the other variables. The statistically significant level was set at *p <* 0.05.
3. Two models of multiple linear and forward stepwise regression were designed by introducing every independent variable, calculating the beta, standard error, and confidence intervals values at 95%. Linearity and independence of residuals were verified by means of the Durbin-Watson statistic, determining acceptable independence values inferior to 2.5.

### Ethical Considerations

This study follows the good clinical practice, as specified by European Directive 2001/20/CE and Law 14/2007, of July 3, on biomedical research. For the treatment of personal data, the provisions of the Spanish Organic Law 3/2018 of December 5 on Protection of Personal Data and Guarantee of Digital Rights were followed. All participants were informed of the objectives of the study and provided their consent to participate by checking a specific box. In addition, the study was authorised by the Regional Hospital management.

## Results

### Characteristics of the Sample

The total number of births during this period was 921. The questionnaire was answered by 83 women. Finally, 14 questionnaires were excluded because they did not comply with the inclusion criteria, so 69 were analysed. The mean age of participants was 30.94 (SD 5). With respect to marital status, 87% were in a relationship, 66.6% were Christians, 76.8% had a higher level of education, and 82.6% were working (Table 1).

**Table 1:** Sociodemographic characteristics of the study sample (*n* = 69)

	**Participants** **M (SD)**
Age (years old)	30.94 (5)
	***n*** (%)
≤ 30 years old	31 (44.9%)
> 30 years old	38 (55.1%)
Relationship status	
Single	9 (13%)
In a relationship	60 (87%)
Religion	
Christianism	46 (66.6%)
Islamism	18 (26.1%)
Atheistic/Agnostic	5 (7.2%)
Level of education	
Basic education	16 (23.2%)
Higher level of education	53 (76.8%)
Working status	
Unemployed	12 (17.4%)
Employed	57 (82.6%)

M = mean; SD = standard deviation; *n* = frequency; % = percentage

The mean of number of children was 1.86 (SD 0.97). Out of the entire sample, 68.1% had attended maternal education classes, with a mean of classes of 4.03 (SD 4.28) and 63.8% had had help to take care of the new-born during the postpartum period. As regards antecedents of mood disorder, 84.1% stated they had had some at some point in their lives (Table 2).

**Table 2:** Clinical variables of the study sample (*n* = 69)

	**M (SD)**
Parity (No. of children)	1.86 (0.97)
No. of maternal education classes	4.03 (4.28)
	***n*** (%)
Nullipara	32 (46.4%)
Multipara	37 (53.6%)
Maternal education	
Yes	47 (68.1%)
No	22 (31.9%)
Help to take care of the new-born during the postpartum period	
Yes	44 (63.8%)
No	25 (36.2%)
Antecedents of mood disorder	
With antecedents	58 (84.1%)
Without antecedents	11(15.9%)

*M* = mean; *SD* = standard deviation; *n* = frequency; % = percentage

Regarding variables related to the pandemic, 78.3% of women had not been diagnosed with COVID-19 and 52.2% had not had a close contact with a person diagnosed with it. As regards the difficulty for the follow-up of pregnancy by the Health Centre during the pandemic, 92.8% of women represented that they had had some difficulty; the most frequently stated one was the fact of not being accompanied to follow-up consultations during pregnancy (Table 3).

**Table 3:** Pandemic-related variables of the study sample (*n* = 69)

	*n* (%)
COVID-19+ diagnosis	
Yes, during pregnancy	9 (13%)
Yes, during the postpartum period	6 (8.7%)
No	54 (78.3%)
Close contact with someone with COVID-19 during pregnancy and/or the postpartum period	
Yes	33 (47.8%)
No	36 (52.2%)
Difficulties in the follow-up of pregnancy during the pandemic	
Yes	64 (92.8%)
No	5 (7.2%)
Stated difficulties	
Not being accompanied in consultations	52 (75.4%)
Delay in testing	32 (46.4%)
Delay in attention	31 (44.9%)
Telephone consultations	31 (44.9%)

*M* = mean; *SD* = standard deviation; *n* = frequency; % = percentage

The mean value according to the Edinburgh scale was 16.14 ± 6.54; 85.5% showed risk of depression. While the postpartum anxiety mean was 26.91 ± 12.32, 40.6% of women showed serious anxiety (Table 4).

**Table 4:** Depression and anxiety levels

	**M (SD)**
Total Depression Risk (Edinburgh Scale)	16.14 (6.54)
Total Anxiety (Stai-E Anxiety Scale)	26.91 (12.32)
	***n*** (%)
Edinburgh Scale	
No PPD risk	10 (14.5%)
PPD risk	59 (85.5%)
STAI-E Anxiety	
No anxiety	25 (36.2%)
Mild anxiety	8 (11.6%)
Moderate anxiety	8 (11.6%)
Serious anxiety	28 (40.6%)

*M* = mean; *SD* = standard deviation; *PPD* = postpartum depression; *n* = frequency; % = percentage

### Bivariate Analysis of Levels of Anxiety and Postpartum Depression

The number of children variable is significantly associated with anxiety, multipara women showing higher levels of anxiety. Having been diagnosed with COVID-19, having had close contact with someone with COVID-19, and having had no help during the postpartum period are closely related to higher levels of anxiety, while having antecedents of mood disorder was related to higher levels of postpartum anxiety and depression (Table 5).

**Table 5:** Bivariate analysis for the classified depression and anxiety scales

	Anxiety					Depression		
	No Anxiety	Mild	Moderate	Serious	*p*	No risk	Risk	*p*
	***n*** (%)						***n*** (%)	
Age								
≤30	15 (21.7%)	5 (7.2%)	5 (7.2%)	6 (8.7%)	0.014*	5 (7.2%)	26 (37.7%)	0.495
>30	10 (14.5%)	3 (4.3%)	3 (4.3%)	22 (31.9%)		5 (7.2%)	33 (47.8%)	
No. of children								
Primipara	17 (24.6%)	4 (5.8%)	7 (10.1%)	4 (5.8%)	0.000**	4 (5.8%)	28 (40.6%)	0.465
Multipara	8 (11.6%)	4 (5.8%)	1 (1.4%)	24 (34.8%)		6 (8.7%)	31 (44.9%)	
Marital Status								
In a relationship	22 (31.9%)	6 (8.7%)	6 (8.7%)	26 (37.7%)	0.407	8 (11.6%)	52 (75.4%)	0.387
Not in a relationship	3 (4.3%)	2 (2.9%)	2 (2.9%)	2 (2.9%)		2 (2.9%)	7 (10.1%)	
Level of education								
Basic level	7 (10.1%)	2 (2.9%)	2 (2.9%)	5 (7.2%)	0.849	2 (2.9%)	14 (20.3%)	0.579
Higher education	18 (26.1%)	6 (8.7%)	6 (8.7%)	23 (33.3%)		8 (11.6%)	45 (65.2%)	
Working Status								
Employed	23 (33.3%)	8 (11.6%)	5 (7.2%)	21 (30.4%)	0.086	9 (13%)	48 (69.6%)	0.444
Unemployed	2 (2.9%)	0 (0%)	3 (4.3%)	7 (10.1%)		1 (1.4%)	11 (15.9%)	
Maternal Education								
Yes	16 (23.2%)	5 (7.2%)	3 (4.3%)	23 (33.3%)	0.098	5 (7.2%)	42 (60.9%)	0.167
No	9 (13%)	3 (4.3%)	5 (7.2%)	5 (7.2%)		5 (7.2%)	17 (24.6%)	
COVID-19 + diagnosis								
Yes	0 (0%)	1 (1.4%)	1 (1.4%)	13 (18.8%)	0.000*	0 (0%)	15 (21.7%)	0.070
No	25 (36.2%)	7 (10.1%)	7 (10.1%)	15 (21.7%)		10 (14.5%)	44 (63.8%)	
Close contact with someone with COVID-19								
Yes	5 (7.2%)	4 (5.8%)	1 (1.4%)	23 (33.3%)	0.000**	4 (5.8%)	29 (42%)	0.425
No	20 (29%)	4 (5.8%)	7 (10.1%)	5 (17.9%)		6 (8.7%)	30 (43.5%)	
Difficulties in the follow-up of pregnancy during the pandemic								
Yes	21 (30.4%)	8 (11.6%)	8 (11.6%)	27 (39.1%)	0.198	10 (14.5%)	54 (78.3%)	0.445
No	4 (5.8%)	0 (0%)	0 (0%)	1 (1.4%)		0 (0%)	5 (7.2%)	
Antecedents of mood disorder								
With antecedents	16 (23.2%)	7 (10.1%)	7 (10.1%)	28 (40.6%)	0.005*	5 (7.2%)	53 (76.8%)	0.007*
Without antecedents	9 (13%)	1 (1.4%)	1 (1.4%)	0 (0%)		5 (7.2%)	6 (8.7%)	
Postpartum help								
Yes	22 (31.9%)	7 (10.1%)	5 (7.2%)	10 (14.5%)	0.000**	8 (11.6%)	36 (52.2%)	0.216
No	3 (4.3%)	1 (1.4%)	3 (4.3%)	18 (26.1%)		2 (2.9%)	23 (33.3%)	

*n* = frequency; % = percentage; * = *p* ≤ 0.05; ** = *p* ≤ 0.01

### Multiple linear regression model

Table 6 shows the final linear regression model for postpartum depression with the two predictor variables that explained the 36.9% of variability. Women who had had mood antecedents before pregnancy and who were diagnosed with COVID-19 during pregnancy or the postpartum period show a higher score in the postpartum depression risk scale.

**Table 6:** Multiple linear regression model for postpartum depression

Variable	*β*	Error Dev.	95% CI	
Mood antecedents	8.421	1.782	4.863	11.978
COVID-19+ diagnosis	4.488	1.581	1.331	7.646

Durbin-Watson Test = 1.577; *F* = 19.288; *p* < 0.000

Table 7 shows the final linear regression model for postpartum anxiety with the three predictor variables that explained the 46.6% of variability. Women who had had mood antecedents before pregnancy, who were diagnosed with COVID-19 during pregnancy/the postpartum period and who have more than one child show a higher anxiety level during the postpartum period.

**Table 7:** Multiple linear regression model for postpartum anxiety

Variable	*β*	Error Dev.	95% CI	
Mood antecedents	14.175	3.157	7.870	20.479
COVID-19+ diagnosis	8.781	2.910	2.970	14.592
Being multipara	5.513	2.407	0.706	10.321

Durbin-Watson Test = 1.845; *F* = 18.889; *p* < 0.000

## Discussion

The purpose of this study was to identify the anxiety level and the postpartum depression risk during the pandemic, related factors, and predictive factors. This study contributes significant data on the influence of COVID-19 on the development of anxiety and postpartum depression risk.

Previous studies conducted before the pandemic reported a mean prevalence of postpartum anxiety of 18.1 ± 7.4, mild anxiety predominating (Gancedo-García et al., 2017). Our results, however, show a higher prevalence of postpartum anxiety during the last year due to the pandemic, with a mean of 26.91 ± 12.32, severe anxiety being the most predominant. The results obtained on the risk of PPD in our sample were an average of 16.14 ± 6.54, while studies carried out before the pandemic (Míguez et al., 2017), informed mean values of 8.16. This data shows that the risk of PPD has increased in the pandemic period. Both results confirm our first hypothesis, which posited that the risk of PPD and/or anxiety differs between those who gave birth before and after the COVID-19 pandemic and confirm that the risk is higher in both cases.

The increase in anxiety and depression risk levels during the pandemic may be due to the several measures taken by the government to reduce the propagation of the disease, including social isolation, which have had a negative impact on the mental health of the population from multiple countries, in general (Le et al., 2020; Wang et al., 2020; Wang et al., 2020; Wang et al., 2021), and on puerperal women, in particular (Galea, Comerciante & Lurie, 2020; Brisk et al., 2021). Additionally, the study conducted by Wang, et al. (2021) found that the Spanish population reported higher levels of stress and depression, as well as more symptoms and use of medical services than participants from China.

Our study wanted to analyse other factors that may influence anxiety disorders and PPD, as established in the initial hypotheses. In accordance with the second hypothesis, the sociodemographic variables such as age, marital status, level of education, working status, and maternal education were not significantly related to the appearance of postpartum anxiety and depression in our sample, as stated by Pham et al. (2017) before the pandemic. Nevertheless, such result differs from other studies, which consider that being younger than 30 and single are PPD risk factors (Cordova, 2017; Meléndez et al., 2017; Wu et al., 2020). While as regards the level of education, several authors disagree with our results by stating that having primary education or being an illiterate person is related to the risk of PPD appearance (Cordova, 2017; Míguez et al., 2017; Pham et al., 2017); in contrast, Yan et al’s study (2020) noted that it was related to the mothers’ high level of education.

Although our results did not show an association between working status and these pathologies, many authors represented that unemployed mothers had higher PPD risk (Míguez et al., 2017; Pham et al., 2017; Romero et al., 2017; Yan et al., 2020). Likewise, having a low socioeconomic level is deemed a risk factor for such disorder (McCartney, 2019). These variables in our study may have been not significant due to the participants’ profile, most of our sample was older than 30, were in a relationship, had a higher level of education, and were employed.

With regard to the third hypothesis (the risk of PPD and/or anxiety differs between women who have help raising their children and those who do not), our study shows an increase in cases of postpartum anxiety and depression risk in women who had no help to take care of the new-born. Therefore, our results confirm this hypothesis and are in accordance with previous research (Brisk et al., 2021; Berthold et al., 2020; Fallon et al., 2021; Pham et al., 2017; Wu et al., 2020; Yan et al., 2020). In our results, limitation on health attendance has had no impact on the increase in cases of such pathologies; however, Davenport et al. (2020) discovered a relationship between health restrictions and an increase in psychological issues; this may be due to the fact that the women of our study accepted the health attention restrictions imposed due to the pandemic.

In response to hypothesis four (the risk of PPD and/or anxiety differs between those who have a history of mood disorders and those who do not), when adjusted to the rest of variables, having mood antecedents maintained an association with postpartum anxiety and depression risk. Hence, this result confirms the hypothesis and coincides with other studies performed before the pandemic (Gancedo-García et al., 2017; Míguez et al., 2017; Pham et al., 2017; Spinola et al., 2020; Brisk et al., 2021). It can be stated that this variable could be considered a risk factor present both in non-pandemic and pandemic contexts.

Regarding hypothesis five (the risk of PPD and/or anxiety differs between new and multiparous mothers), our results show that the number of children was also significantly related to both pathologies. Multipara women reported more postpartum anxiety and risk of depression. These results confirm this hypothesis and are in line with other authors (Romero et al., 2017; Bertholdt et al., 2020; Yan et al., 2020). This is justified on the basis that taking care of a new-born is a great psychological burden that alters the mother’s mental health, such disorder being higher during the pandemic as a consequence of the social isolation they had to face and not being able to receive any help with the baby from their relatives and/or acquaintances, in addition to taking care of the other children not attending school or doing so within reduced hours.

When considering COVID-related variables, those who had close contact with the disease and/or a positive diagnosis during pregnancy or the postpartum period showed more anxiety and risk of PPD. This finding coincides with the results of Wu et al.’s (2020) and Missler et al.’s studies (2020). This may be due to the fear pregnant women felt about the lack of knowledge on the vertical transmission to the foetus (Romero-González et al., 2020; Spinola et al.,^2020^). These results confirm our last hypothesis, the risk of suffering PPD and/or anxiety differs among those who have maintained close contact with or have been infected by COVID-19.

Online psychological interventions such as cognitive behavioural therapy (CBT) or mindfulness based cognitive therapy (MBCT) could have been useful in relieving stress and reducing postpartum anxiety (Ho, Chee & Ho, 2020; Soh et al., 2020; Zhang & Ho, 2017). In addition, accurate dissemination of health information and follow-up during pregnancy and postpartum by telephone or online would reduce depression during this period.

## Limitations

This study has several limitations that should be acknowledged. First, the sample size is small, so that the results are not extrapolated. Second, during the pandemic the birth rate in the city decreased due to mobility reasons derived from the geographical characteristics of Melilla, an isolated border city. In addition, access to Health Centres to recruit women was restricted. In order to reduce this limitation, successive calls were made at different times. Finally, this study was not randomised, therefore it is possible that respondents were the most motivated women with more knowledge of such variables; nonetheless, given the pandemic context and the limited access to women, a randomised sample was not a choice.

## Conclusion

Almost half of the women of our study who gave birth during the pandemic showed serious anxiety. This was higher in multiparous women, who had had close contact with the disease, who had been diagnosed with COVID-19, with antecedents of mood disorder, and who had no help during the postpartum period to take care of the new-born. Most of the sample showed high levels of PPD risk during such period. Depression risk was higher in puerperal women who had had close contact with COVID-19 patients, who had been diagnosed with it, who had mood antecedents and who had no help to take care of the baby. Mood antecedents and a positive COVID-19 diagnosis during pregnancy or the postpartum period predicted an increase in the risk of postpartum depression; while for postpartum anxiety, the predictive factor of being multipara must be added to mood antecedents and being diagnosed with COVID-19.

## Supplementary Information

ESM 1 STROBE Statement—checklist of items that should be included in reports of observational studies (DOCX 28 kb)

## Data Availability

The data that support the findings of this study are available from the corresponding author upon reasonable request.
